# CD200–CD200R as a myeloid immune checkpoint in solid tumors: mechanisms, context-dependent functions, and therapeutic opportunities

**DOI:** 10.3389/fimmu.2026.1950382

**Published:** 2026-09-18

**Authors:** Manas Ranjan Sahu, Inemai Ezhil, Venu Akkanapally, Aman Kumar, Jin-Qing Liu, Sujit Basu

**Affiliations:** 1 Department of Pathology, Ohio State University, Columbus, OH, United States; 2 Division of Medical Oncology, Department of Internal Medicine, Ohio State University, Columbus, OH, United States; 3 Comprehensive Cancer Center, Ohio State University, Columbus, OH, United States

**Keywords:** CD200, CD200R, solid tumors, therapy, tumor microenvironment

## Abstract

CD200 and its receptor CD200R form a myeloid-centered immune checkpoint that normally restrains myeloid activation and limits tissue damage, but many solid tumors co-opt this pathway to suppress antitumor immunity. CD200–CD200R signaling is highly context-dependent, varying even among models of the same cancer type. In melanoma and breast carcinoma, it can either suppress protumor inflammation or promote tumor growth and metastasis depending on the model, whereas in neuroblastoma; glioblastoma and other central nervous system tumors; cutaneous and head and neck squamous cell carcinoma; colorectal cancer; and several endocrine, ovarian, and pancreatic cancers, it consistently suppresses macrophage, dendritic cell, natural killer cell, and CD8+ T-cell activation, impairs phagocytosis and antigen presentation, and drives immunosuppressive infiltration. Non-small cell lung cancer and renal cell carcinoma emerge as two additional, less-characterized but clinically important contexts warranting further investigation. CD200R1–CD200 has also recently been identified as a macrophage phagocytosis checkpoint that functions independently of CD47–SIRPα, placing it within the expanding family of innate immune checkpoints. Rather than treating CD200–CD200R as a universally protumorigenic target, we propose a context-dependent framework—incorporating tumor type, microenvironmental architecture, and dominant myeloid and lymphoid programs—to guide tumor-specific therapeutic strategies in solid cancers.

## Introduction

Immune checkpoint blockade targeting programmed cell death protein 1/programmed death-ligand 1 (PD-1/PD-L1) and cytotoxic T-lymphocyte-associated protein 4 (CTLA-4) has transformed the treatment of several solid tumors; yet most patients either fail to respond or eventually develop disease progression. One major limitation is that these T-cell-directed therapies do not adequately address the myeloid-driven immunosuppression that characterizes many tumor microenvironments (1, 2). This unmet need has driven increasing interest in myeloid immune checkpoints—inhibitory pathways expressed on macrophages, dendritic cells, and other innate immune cells—as complementary therapeutic targets, with the CD47–SIRPα axis representing the most clinically advanced example to date (3, 4).

CD200 and its receptor CD200R were originally identified as regulators of peripheral immune tolerance and tissue protection during inflammatory responses (5–7). Over the past two decades, increasing evidence has revealed that many solid tumors exploit this tolerogenic pathway to suppress antitumor immunity (8). More recently, the CD200R1–CD200 axis has also been recognized as a macrophage phagocytosis checkpoint independent of CD47–SIRPα (9, 10).

Unlike most other myeloid immune checkpoints, CD200–CD200R signaling is highly context-dependent: specifically, depending on tumor type, it may either promote immune evasion or suppress protumor inflammation (11–14). This review first outlines the molecular biology of the CD200–CD200R pathway and then examines its tumor-specific roles in melanoma, breast carcinoma, central nervous system (CNS) tumors, neuroblastoma, squamous cell carcinomas, colorectal cancer, and endocrine, ovarian, and pancreatic malignancies; additionally, it considers non-small cell lung cancer (NSCLC) and renal cell carcinoma (RCC) as emerging, clinically important but currently underexplored contexts before discussing its emerging role as a phagocytosis checkpoint, as well as its therapeutic and biomarker implications.

The CD200–CD200R axis has previously been reviewed as a bidirectional immune checkpoint across hematologic and solid malignancies (15) and as an independent therapeutic target (16). Rather than revisiting these broader perspectives, this review focuses exclusively on solid tumors, excluding hematologic malignancies, to develop a tumor-specific framework for understanding the biological and therapeutic roles of the CD200–CD200R axis in solid cancers. This review differs from previous work in three key aspects. First, whereas earlier reviews primarily organize the evidence according to immunological mechanisms—for example, natural killer (NK) cell suppression, regulatory T-cell (Treg) expansion, or recruitment of myeloid-derived suppressor cells (MDSCs)—we adopt a tumor-centered approach. Specifically, we examine how the same signaling pathway produces divergent, and in some cases model-dependent, effects across different solid tumors, including melanoma, breast carcinoma, neuroblastoma, glioblastoma, squamous cell carcinoma, and colorectal cancer. This framework enables us to relate the biological role of the CD200–CD200R axis within each tumor type to its distinct therapeutic implications, rather than proposing a single, uniform therapeutic strategy. Second, we incorporate recent mechanistic and clinical advances that have emerged since those earlier reviews, including the identification of the CD200R1–CD200 axis as a macrophage phagocytosis checkpoint that functions independently of CD47–SIRPα (9) and the first-in-human clinical evaluation of the CD200R1-blocking antibody 23ME-00610 (17). These developments enable us to position the CD200–CD200R axis within a comparative framework of innate immune checkpoints alongside CD47–SIRPα, an approach that, to our knowledge, has not previously been presented in a review focused exclusively on solid tumors (3). Third, we extend beyond describing the underlying biology by translating tumor-specific mechanisms into potential combination therapeutic strategies and a biomarker-guided framework for patient selection. This clinically oriented perspective is intended to support the rational design of future clinical trials.

## CD200–CD200R biology and myeloid regulation

CD200 is a type I transmembrane glycoprotein belonging to the immunoglobulin superfamily. It consists of two extracellular immunoglobulin-like domains and a short cytoplasmic tail that lacks canonical signaling motifs (7). CD200 is widely expressed on activated T and B cells, dendritic cells, endothelial and epithelial cells, neurons, and many solid tumor cells. Its expression can be further upregulated by inflammatory mediators, including interferon-γ (IFN-γ) and tumor necrosis factor (TNF) (7, 18, 19). In addition to the membrane-bound form, a soluble form generated by shedding retains the ability to bind CD200R and may extend immunosuppressive signaling beyond the local tumor microenvironment (20).

**Figure 1:**
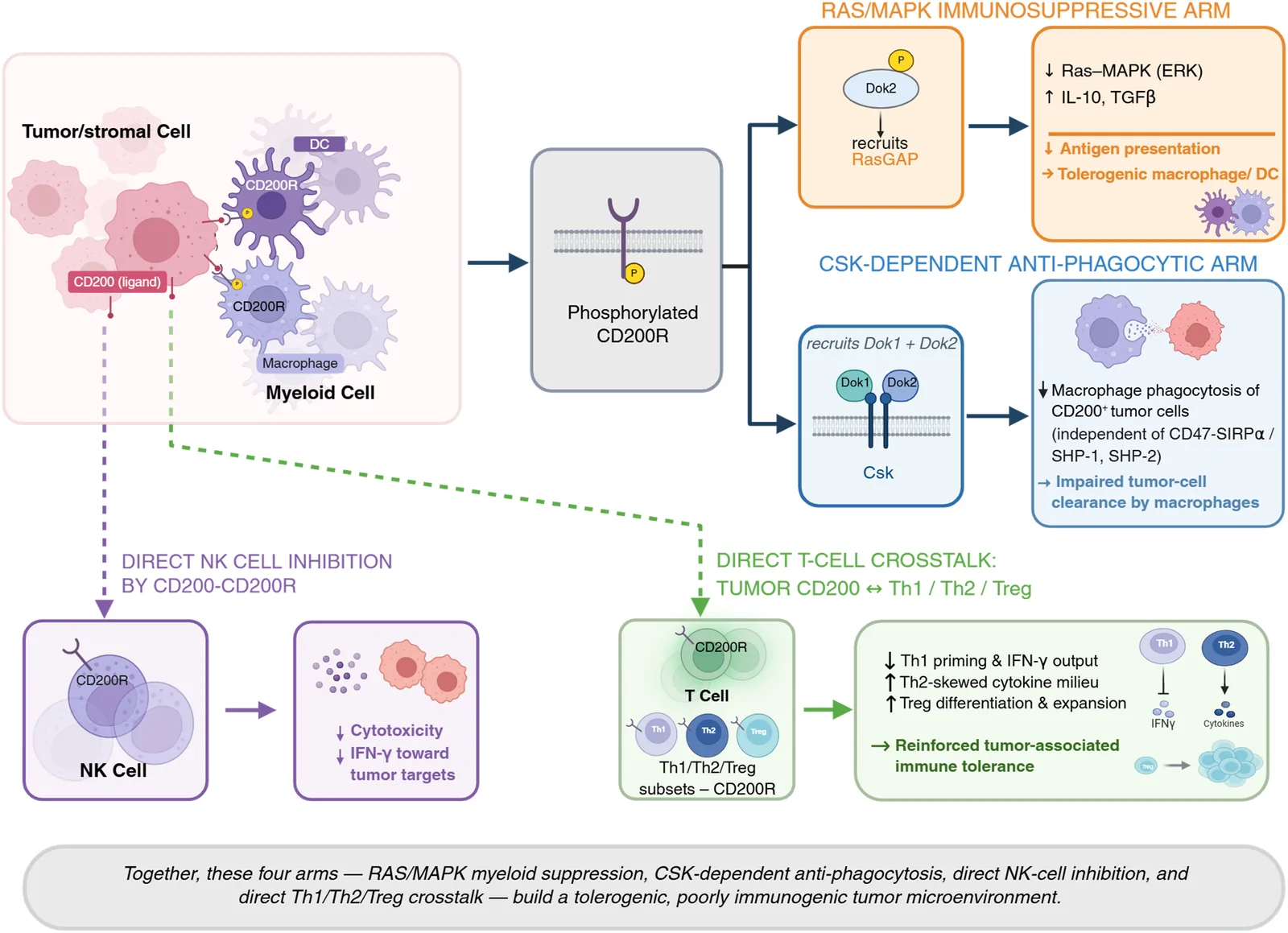
CD200 biology and CD200–CD200R signaling in myeloid regulation. CD200 expressed on tumor or stromal cells binds to CD200R on macrophages and dendritic cells (DC), thereby inducing receptor phosphorylation and the recruitment of Dok2. Dok2 recruits Ras GTPase-activating protein (RasGAP), which suppresses Ras/MAPK signaling, increases IL-10/TGF-β production, and promotes a tolerogenic myeloid phenotype with reduced antigen presentation. Through a distinct signaling branch, Dok1 and Dok2 recruit Csk, thus suppressing macrophage phagocytosis of CD200^+^ tumor cells independent of the CD47–SIRPα/SHP-1/SHP-2 pathway. CD200–CD200R signaling also directly inhibits NK-cell cytotoxicity and IFN-γ production. In parallel, tumor CD200 directly engages CD200R on T-cell subsets, thereby restraining Th1 priming and IFN-γ output, favoring a Th2-skewed cytokine milieu, and promoting Treg differentiation and expansion, which reinforces tissue- and tumor-associated immune tolerance. Collectively, these four arms establish an immunosuppressive, poorly immunogenic tumor microenvironment. The image was created using BioRender.com.

CD200R1 is the principal functional receptor for CD200 in humans and is expressed on monocytes, macrophages, dendritic cells, granulocytes, and subsets of NK and T cells (21–23). Binding of CD200 to CD200R1 induces receptor phosphorylation and recruitment of the adaptor protein downstream of kinase 2 (Dok2), which subsequently recruits Ras GTPase-activating protein (RasGAP) to suppress Ras–mitogen-activated protein kinase (MAPK) signaling (24). Collectively, these effects promote tolerogenic phenotypes in macrophages and dendritic cells, characterized by increased interleukin-10 (IL-10) and transforming growth factor-β (TGF-β) production and reduced antigen presentation (5, 19). In NK cells, CD200–CD200R signaling also directly inhibits cytotoxic activity and IFN-γ production in response to tumor cells (22) (**Figure 1**).

In parallel to these myeloid-directed effects, tumor-expressed CD200 engages CD200R present directly on T-cell subsets, including Th1, Th2, and Treg populations, providing an antigen-presenting-cell-independent mechanism of immune regulation within the tumor microenvironment. This direct CD200–CD200R interaction restrains Th1 priming and IFN-γ output, favors a Th2-skewed cytokine milieu, and promotes Treg differentiation and expansion, thereby reinforcing tissue- and tumor-associated immune tolerance (19, 25, 26). Consistent with this direct T-cell axis, elevated tumor CD200 expression in solid tumor models and human solid tumors is correlated with an expanded FoxP3^+^ Treg compartment alongside reduced Th1 cytokine output, supporting the contribution of this pathway to a tolerogenic tumor microenvironment, independent of myeloid antigen presentation (15, 27, 28). Together with CD200–CD200R-mediated suppression of myeloid antigen presentation and NK-cell cytotoxicity, this direct T-cell crosstalk constitutes a distinct arm of CD200–CD200R-mediated immune regulation, as depicted in **Figure 1**.

Under physiological conditions, this pathway functions as a protective mechanism that limits excessive myeloid activation and tissue inflammation rather than representing an immunologic defect. CD200-deficient mice exhibit exaggerated inflammatory responses, particularly in the CNS and lungs, and activation of CD200R promotes Treg differentiation as part of normal tissue tolerance and immune homeostasis (5, 26, 29). Tumors arising in tissues with constitutively active CD200-mediated immune regulation, such as the brain and skin, appear to exploit this homeostatic pathway to establish an immunosuppressive microenvironment (30, 31).

## CD200–CD200R in solid tumor microenvironments

### General features across solid tumors

Across solid tumors, CD200 expressed by tumor or stromal cells engages CD200R on tumor-associated macrophages, MDSCs, and dendritic cells, thereby suppressing their activation and antigen-presenting capacity (15, 32, 33). These CD200R-expressing myeloid cells subsequently remodel the local chemokine milieu, promoting the recruitment of Tregs and other immunosuppressive cell populations while limiting the infiltration and activity of effector T cells and NK cells (12, 34). In addition, soluble CD200 (sCD200) generated through shedding can extend immunosuppressive signaling beyond the primary tumor, particularly in the presence of a high circulating tumor burden or extensive stromal shedding (20, 35). Collectively, these mechanisms drive the myeloid compartment toward an immunoregulatory phenotype rather than effective antitumor immunity. Although this general pattern has been described previously (36), its biological and therapeutic consequences differ substantially among solid tumor types.

### Melanoma: a model-dependent, occasionally tumor-restraining context

Melanoma provides some of the strongest evidence that CD200–CD200R signaling can suppress, rather than promote, tumor progression. In B16 melanoma models, tumor cells engineered to express CD200 exhibited reduced tumor formation and fewer lung metastases than CD200-negative controls, an effect that was abolished in CD200R-deficient hosts (13, 37). Mechanistically, activation of CD200R on myeloid cells suppresses protumor inflammatory responses, limits vascular endothelial growth factor- and hypoxia-inducible factor (HIF)-mediated angiogenesis, and reduces the recruitment of tumor-promoting myeloid populations (13). In addition, tumor-derived CD200 has been shown to suppress IL-10 production by tumor-associated myeloid cells, promoting a shift toward an M1-like macrophage phenotype and enhancing the efficacy of adoptive cytotoxic T-lymphocyte therapy (38).

When CD200 blockade was evaluated in the Yumm1.7 murine melanoma model, it altered the tumor immune microenvironment but failed to inhibit tumor growth (14), underscoring that CD200 blockade is not uniformly beneficial across melanoma models.

However, findings reported by Lin et al. further highlight the complexity of CD200–CD200R signaling in melanoma. In CD200R-deficient mice bearing CD200-expressing Yummer1.7 tumors, tumor growth was significantly slower than in wild-type controls and was accompanied by increased infiltration of CD4+ T cells, CD8+ T cells, and NK cells, reduced neutrophil infiltration, and transcriptional reprogramming of tumor-associated myeloid cells (12). Specifically, CD200R deficiency increased expression of the eosinophil-recruiting chemokine CCL24 while reducing expression of the neutrophil-associated chemokines CXCL2, CXCL3, and CCL3 through extracellular signal-regulated kinase (ERK)/p38 signaling (12). These findings contrast with the tumor-restraining effects observed in B16 melanoma models. The authors proposed that this discrepancy may reflect differences in tumor immunogenicity or context-dependent functions of CD200R across distinct immune cell populations (12). Collectively, these findings indicate that the biological effects of CD200–CD200R signaling in melanoma are highly model-dependent rather than uniformly tumor-restraining. Consequently, therapeutic strategies aimed at either blocking or enhancing this pathway should be guided by tumor-specific biological context until its overall role in human melanoma is more clearly defined.

### Breast carcinoma: another model-dependent context

A similar context-dependent pattern is observed in breast carcinoma. In an aggressive 4THM murine breast carcinoma model, host overexpression of CD200 reduced neutrophil infiltration and the production of TNF-α and IL-6, resulting in decreased visceral metastasis. Conversely, loss of CD200R1 increased metastatic spread, supporting a tumor-restraining role for CD200–CD200R signaling in this model (39).

However, earlier genetic loss-of-function studies from the same research group challenge this interpretation. Podnos et al. reported that CD200R1-knockout mice exhibited substantially reduced EMT6 tumor growth and markedly lower lymph node metastasis than wild-type recipients, irrespective of tumor CD200 expression. Similarly, CD200-silenced EMT6 cells failed to metastasize unless rescued by co-injection with CD200-overexpressing cells, whereas CD200-transgenic hosts supported greater metastatic dissemination (40). Both CD200R1-deficient mice and mice bearing CD200-silenced tumors generated stronger antitumor cytotoxic responses, and adoptive transfer of lymph node cells from these mice suppressed tumor growth in secondary recipients (40). Collectively, these findings contrast with those of the 4THM model and indicate that, in the EMT6 model, interactions between tumor- and host-derived CD200 and CD200R1 promote, rather than suppress, metastatic progression.

### Neuroblastoma: a protumor myeloid immune checkpoint

In contrast to melanoma and selected breast carcinoma models, neuroblastoma exemplifies the protumor role of the CD200–CD200R axis. Neuroblastoma cells overexpress CD200, and engagement of CD200R on tumor-associated myeloid cells alters chemokine production to establish an immunosuppressive tumor microenvironment and reduce tumor rejection in murine models (12, 41). Consequently, tumor-infiltrating T cells exhibit diminished IFN-γ and TNF-α production and remain in a functionally restrained, rather than fully exhausted state, consistent with chemokine-mediated exclusion of effector immune cells (12, 41). Given the strong dependence of neuroblastoma on myeloid and stromal support, the CD200–CD200R pathway represents a promising therapeutic target for combination strategies with established treatment modalities, including anti-GD2 antibody therapy, PD-1 blockade, and myeloid-reprogramming approaches (42).

### Glioblastoma and CNS tumors: local immune tolerance and resistance to immunotherapy

CNS tumors, particularly glioblastoma, exemplify the role of CD200–CD200R signaling in maintaining local immune tolerance. Both tumor cells and the surrounding neural tissue express CD200, and engagement of CD200R suppresses microglial and macrophage activation, thereby contributing to the profound immunosuppressive microenvironment that characterizes glioblastoma and limits therapeutic efficacy (31). Consistent with this mechanism, blockade of the CD200 pathway enhances vaccine efficacy and T-cell infiltration in preclinical glioma models (31).

These findings support evaluation of CD200 pathway inhibition in combination with glioma vaccines, oncolytic virotherapy, or myeloid-reprogramming approaches such as colony-stimulating factor 1 receptor (CSF1R) inhibitors, which promote macrophage repolarization and suppress glioma progression (43). CD200 blockade may also complement cellular immunotherapies, including chimeric antigen receptor (CAR) T-cell therapy (44). However, because CD200 plays a critical role in maintaining CNS immune homeostasis, clinical studies should incorporate careful monitoring for neurological and inflammatory toxicities.

### Cutaneous and head and neck squamous cell carcinoma: myeloid-driven tumor invasion

Cutaneous and head and neck squamous cell carcinomas represent a more conventional protumor role for the CD200–CD200R axis. In cutaneous squamous cell carcinoma, CD200–CD200R signaling upregulates cathepsin K expression in tumor-infiltrating myeloid cells, promoting degradation of type I collagen, a key component of the extracellular matrix that normally restricts tumor invasion. This process facilitates local invasion and metastatic dissemination (45). Similarly, head and neck squamous cell carcinoma is characterized by a myeloid-rich immunosuppressive tumor microenvironment (46), in which CD200R signaling promotes a chemokine milieu that favors suppressive myeloid cell populations (47) and Tregs while limiting antitumor effector immune response (48).

These findings support evaluation of CD200 or CD200R inhibition in combination with chemoradiotherapy; PD-1 blockade, which is already approved for recurrent and metastatic head and neck squamous cell carcinoma (49); or therapies targeting the CD47–SIRPα phagocytosis checkpoint. Biomarkers such as myeloid infiltration, cathepsin K expression, and local T-cell activation may provide useful indicators of therapeutic response.

### Colorectal cancer: CD200R as a checkpoint on tumor-infiltrating lymphocytes

Preclinical studies using the MC38 colorectal cancer model provide direct evidence that CD200R can function as an inhibitory checkpoint on cytotoxic lymphocytes. Zhang et al. demonstrated that CD200R was upregulated on tumor-infiltrating NK cells and CD8+ T cells, where its expression was associated with an exhausted phenotype characterized by increased expression of CD96, T-cell immunoreceptor with immunoglobulin and ITIM domains (TIGIT), NKG2A, lymphocyte activation gene 3 (LAG3), PD-1, and T-cell immunoglobulin and mucin domain-containing protein 3 (TIM-3). Both germline *Cd200r* deletion and treatment with a blocking anti-CD200R antibody inhibited tumor growth, prolonged survival, and restored NK-cell and CD8+ T-cell function in a manner dependent on both immune cell populations. Furthermore, anti-CD200R therapy synergized with PD-1 blockade, and a human CD200R-blocking antibody similarly suppressed the growth of CD200-positive human colorectal cancer xenografts by enhancing NK-cell cytotoxicity (50).

However, an independent study using the same MC38 model reported that treatment with the CD200R antagonist OX108 alone did not significantly affect tumor growth or survival, despite CD200 expression by MC38 cells. Although combining OX108 with PD-1 blockade showed a trend toward improved complete response rates, the effect did not reach statistical significance and was attributed to potential differences in antibody affinity or CD200 expression among MC38 sublines (51). Taken together, these findings suggest that CD200R functions as an inhibitory checkpoint that limits NK cell- and CD8+ T-cell-mediated antitumor immunity in colorectal cancer. However, the therapeutic efficacy of CD200R blockade may depend on factors such as antibody potency and the selection of patients with CD200-expressing tumors.

The apparent discrepancy between the two studies on MC38 warrants a more thorough examination, as it has direct implications for the clinical translation of CD200R-targeted antibodies. Several non-mutually exclusive explanations may account for the divergent outcomes. First, as directly supported by the studies themselves, antibody-intrinsic properties, including binding affinity, were not equivalent between the antibodies used in the two studies; specifically, the authors of the study reporting negative findings explicitly proposed differences in receptor occupancy or affinity as a likely explanation for the lack of efficacy with OX108 monotherapy (51). Second, the inconsistent efficacy of CD200R blockade may partly reflect differences in experimental design. The study reporting a greater benefit from CD200R blockade used a 10-fold lower MC38 inoculum, initiated treatment at earlier points in time, and administered a more intensive regimen of five doses. In contrast, the study demonstrating a lower efficacy of OX108-mediated CD200R blockade treated established tumors with only four doses and observed minimal therapeutic benefit. These differences may have created a more favorable setting in the former study for detecting anti-CD200R activity. Third, notably, in the study reporting positive findings, both germline *Cd200r* deletion and antibody-mediated CD200R blockade were tested as independent interventions, and each scenario produced a therapeutic benefit, thereby arguing against genetic-versus-pharmacologic differences as a major driver of the discrepancy between the two studies and instead reinforcing antibody quality and target expression level as the more likely explanations (50).

These considerations provide direct guidance for the design of clinical-grade CD200R-blocking antibodies. First, rigorous head-to-head affinity and receptor-occupancy characterization should be incorporated at early points in time in antibody development, as suboptimal affinity may be sufficient to explain therapeutic failure even when the target pathway is biologically relevant. Second, because the tumor CD200 expression level appears to modulate the magnitude of the achievable benefit, preclinical efficacy data should be interpreted in the context of the CD200 expression status of the specifically utilized tumor model; where possible, the data should be corroborated across independently derived tumor sublines. Third, given the sensitivity of therapeutic outcomes to CD200 expression levels and antibody potency, early-phase clinical trials of CD200R-blocking antibodies should incorporate biomarker-driven patient selection, prospective confirmation of target engagement, and dose-escalation strategies informed by receptor-occupancy pharmacodynamics. Finally, direct comparative testing of candidate antibodies against a shared, well-characterized tumor model and defined CD200 expression threshold would help resolve whether the discrepancy between these studies on MC38 reflects a true biological inconsistency or is instead attributable to differences in antibody quality and target expression; furthermore, the testing would provide clearer preclinical benchmarks to guide the selection and advancement of candidate molecules for clinical development.

### Endocrine, ovarian, pancreatic, and other solid tumors

CD200 is overexpressed in several additional solid tumors, including endocrine malignancies, ovarian cancer, and pancreatic cancer (52–55). Among these malignancies, pancreatic cancer has the strongest functional evidence supporting a role for the CD200–CD200R axis. Multiplex spatial analyses have demonstrated colocalization of CD200 with tumor-infiltrating lymphocytes and cancer-associated fibroblasts, consistent with a localized immunosuppressive microenvironment (54). Patients with pancreatic ductal adenocarcinoma exhibit elevated CD200R expression on MDSCs, and preclinical studies have shown that CD200 blockade suppresses tumor growth while enhancing the antitumor efficacy of PD-1 blockade (52). In other tumor types, mechanistic evidence remains less extensive than that available for melanoma or squamous cell carcinoma. Nevertheless, the recurring pattern of impaired macrophage and dendritic cell activation accompanied by increased immunosuppressive immune infiltrates supports the CD200–CD200R axis as a promising myeloid immune checkpoint, particularly in tumors in which stromal–myeloid interactions are major determinants of disease progression.

### Non-small cell lung cancer: an underexplored context with high clinical relevance

Given that immune checkpoint inhibitors are among the most widely used systemic therapies in NSCLC, the CD200–CD200R axis warrants more detailed consideration in this tumor type than that reported previously. Quantitative expression analyses have revealed that both CD200 and CD200R are detectable on tumor cells and tumor-infiltrating immune cells across NSCLC cohorts, with substantial heterogeneity in expression levels and localization being observed among patients and even within individual tumors (56, 57). This heterogeneity mirrors the pattern described for CD200 in pancreatic cancer, which suggests that, as observed in those tumor types, tissue-based CD200/CD200R assessment alone may not fully capture pathway activity that is relevant to therapeutic responses (54, 57).

Mechanistically, direct functional studies of CD200–CD200R signaling in the NSCLC tumor microenvironment remain limited relative to those in melanoma, glioblastoma, or pancreatic cancer. However, NSCLC tumors are characteristically infiltrated by abundant tumor-associated macrophages and MDSCs; as detailed earlier in this review, these populations are the principal effectors of CD200-mediated immunosuppression in other solid tumors through impaired phagocytic and antigen-presenting capacity, thereby skewing toward an immunoregulatory phenotype, as well as the suppression of NK-cell and cytotoxic T-cell function. Analogous to these established mechanisms, CD200 expressed by NSCLC tumor cells is expected to engage CD200R on this abundant myeloid infiltrate to reinforce an immunosuppressive microenvironment; however, this hypothesis has not yet been directly tested in NSCLC-specific functional or ex vivo models, thus representing a significant and clinically important research gap given the central role of the tumor type in immune checkpoint inhibitor therapy.

From a therapeutic perspective, NSCLC represents a particularly compelling setting in which to evaluate CD200–CD200R-targeted agents. A substantial proportion of patients with NSCLC either fail to respond to PD-1/PD-L1 blockade or develop acquired resistance, and myeloid-driven immunosuppression is increasingly recognized as a major contributor to both primary and acquired resistance in this disease (56, 58). Because CD200–CD200R signaling operates in parallel with T-cell-directed checkpoints, the combination of CD200 or CD200R blockade with PD-1/PD-L1 inhibition represents a rational strategy to convert immunologically cold or myeloid-excluded NSCLC tumors into more immunotherapy-responsive phenotypes, which is analogous to the combination strategies that have already been proposed for pancreatic and squamous cell carcinomas in this review. The observation that the CD200R1-blocking antibody 23ME-00610 is well tolerated and achieves dose-dependent target engagement in patients with advanced solid tumors provides a foundation for extending both this approach and similar approaches to biomarker-selected NSCLC populations, with high tumor CD200 expression proposed as a predictive biomarker (17). Future studies should prioritize the mechanistic characterization of CD200–CD200R signaling within NSCLC-specific myeloid subsets, spatial profiling to establish an association between CD200/CD200R expression and functional immune exclusion, and early-phase combination trials with PD-1/PD-L1 inhibitors in this high-need patient population.

### Renal cell carcinoma: an emerging but underexplored context

RCC is a particularly important indication in which the CD200–CD200R axis should be considered, given that immune checkpoint inhibitors, which are mainly administered in combination with antiangiogenic agents, now constitute the standard of care for advanced disease (59). Early immunohistochemical and cell line studies have demonstrated that the CD200 protein is expressed on clear cell RCC tumor cells and on a subset of RCC lines, with staining also being observed in adjacent normal kidney tissue (55). This expression pattern parallels the tissue heterogeneity described for CD200 in other solid tumors throughout this review and indicates that RCC represents a biologically plausible, but currently underexplored, context for CD200-directed biomarkers and therapeutic development.

Mechanistically, the most direct evidence for CD200-mediated immune evasion via NK-cell suppression is derived from research conducted in cutaneous basal cell carcinoma, in which membrane CD200 undergoes ectodomain shedding via matrix metalloproteinases to generate sCD200 within the tumor microenvironment. Moreover, the engagement of CD200R by both membrane-bound and shed CD200 has been observed to suppress NK-cell MAPK signaling, impair NK-cell-mediated cytotoxicity and IFN-γ release, and additionally trigger Fas/FasL-dependent apoptosis of NK cells through the loss of ERK-mediated repression of PPARγ-regulated gene transcription, with the authors proposing that this mechanism may extend to other CD200-expressing cancer types (35). Whether an analogous shedding-dependent, NK-cell-suppressive mechanism operates in RCC has not yet been directly tested. Notably, ADAM9, a metalloproteinase that is capable of mediating the ectodomain shedding of transmembrane proteins, is independently overexpressed in RCC and has been observed to be associated with higher tumor grade and adverse outcomes (60), thus suggesting the hypothesis that ADAM-family sheddases can similarly generate sCD200 within the RCC tumor microenvironment, which remains to be formally tested. Together, these observations suggest that RCC may plausibly share the membrane-bound and sCD200 immunosuppressive mechanisms described elsewhere in this review, potentially with an NK-cell-centered emphasis given the established importance of NK cells in RCC immunosurveillance, which would distinguish this mechanism from the T-cell- and myeloid-centered mechanisms that predominantly occur in melanoma, colorectal cancer, and squamous cell carcinomas. However, direct confirmation of this hypothesis in RCC-specific models is still needed.

From a therapeutic standpoint, RCC represents an especially compelling setting for evaluating CD200–CD200R-targeted therapy. Despite the establishment of PD-1/PD-L1-based combination regimens as a first-line therapy, a substantial proportion of patients with advanced RCC do not achieve durable responses, and resistance to T-cell-directed checkpoint blockade remains a major clinical challenge (59). Because NK cells substantially contribute to antitumor immunity in RCC (61), as well as the fact that CD200-mediated NK-cell suppression has been directly demonstrated in at least one other solid tumor type (35), CD200 or CD200R blockade could plausibly restore the cytotoxic function of NK cells in RCC in a manner that is complementary to, rather than redundant with, existing PD-1/PD-L1-based regimens; this scenario could be of particular value in tumors with relatively limited T-cell infiltration. Given the existing immunohistochemical evidence of CD200 expression in RCC, the clinical maturity of anti-CD200R agents such as 23ME-00610, and the unmet need for additional mechanisms of action beyond T-cell checkpoint blockade in this disease, future studies should prioritize direct assessments of the shedding and NK-cell-suppressive mechanisms of CD200 within RCC-specific models, along with defining the relationship between tissue and circulating CD200 in this tumor type and evaluating CD200R blockade both as a monotherapy in NK-cell-rich, T-cell-poor tumors and as a combination therapy involving existing checkpoint inhibitor-based regimens.

From a clinical perspective, CD200 pathway modulation warrants investigation as an adjunct to established treatment regimens. Potential combination strategies include platinum-based chemotherapy and poly (ADP-ribose) polymerase (PARP) inhibitors in ovarian cancer, as well as chemoradiotherapy and PD-1 blockade in pancreatic cancer. These approaches are supported by growing evidence that myeloid-driven immune regulation plays a central role in determining therapeutic response and treatment resistance in these malignancies.

## CD200R1–CD200 as a phagocytosis checkpoint

Recent studies have established the CD200R1–CD200 axis as a macrophage phagocytosis checkpoint that functions independently of CD47–SIRPα. Engagement of CD200R1 suppresses macrophage phagocytic activity and tumor cell clearance, whereas genetic deletion or therapeutic blockade of CD200R1 or CD200 restores phagocytosis and inhibits tumor growth in preclinical solid tumor models (9). These findings identify CD200–CD200R as a key component of the expanding network of innate immune checkpoints that regulate tumor cell clearance. In this context, the CD200–CD200R axis complements the well-characterized CD47–SIRPα pathway, the prototypical phagocytosis checkpoint. CD47–SIRPα has been extensively validated in solid tumor models (4), shown to enhance the efficacy of antibody-based therapies in hematologic malignancies (62), and demonstrated to promote downstream antitumor T-cell responses following checkpoint blockade (63). Together, these observations position CD200–CD200R and CD47–SIRPα as complementary regulators of macrophage-mediated tumor clearance and provide a rationale for comparative and combination therapeutic strategies targeting innate immune checkpoints.

Compared with the CD47–SIRPα axis, which primarily delivers an inhibitory signal that limits phagocytosis, the CD200R1–CD200 pathway exerts broader immunoregulatory effects. In addition to suppressing macrophage-mediated phagocytosis, it extensively modulates myeloid cell activation and cytokine production (9). Importantly, the phagocytosis-inhibitory function of CD200R1 is mediated through a signaling pathway that is distinct from the canonical Dok2–RasGAP–Ras MAPK cascade described above. Instead, this effect depends on recruitment of the kinase Csk downstream of Dok1 and Dok2 and is independent of the SHP1- and SHP2-mediated signaling mechanisms used by SIRPα (9). The coexistence of these distinct signaling branches—a dedicated Csk-dependent pathway that suppresses phagocytosis together with a Ras/MAPK-dependent pathway that broadly restrains myeloid activation—may explain the potent immunosuppressive capacity of the CD200–CD200R axis. By simultaneously limiting tumor cell engulfment and promoting a tolerogenic myeloid microenvironment, this pathway reduces both innate immune clearance and the subsequent recruitment and activation of antitumor effector cells (**Figure 1**).

### CD200–CD200R within the broader landscape of myeloid immune checkpoints

Beyond CD47–SIRPα, the CD200–CD200R axis should be understood as representing one member of a rapidly expanding family of myeloid immune checkpoints that regulate macrophage and dendritic cell function through mechanistically distinct ligand–receptor pairs. CD24, which is a glycosylphosphatidylinositol-anchored protein that is overexpressed on many solid tumors, engages the inhibitory receptor Siglec-10 on tumor-associated macrophages to deliver a potent antiphagocytic “don’t eat me” signal; additionally, the genetic or antibody-mediated blockade of the CD24–Siglec-10 interaction restores macrophage-mediated clearance of CD24-expressing tumors, with the strongest evidence to date being reported in ovarian and breast cancer (64). The leukocyte immunoglobulin-like receptor B (LILRB) family constitutes a second major class of myeloid inhibitory receptors; specifically, LILRB1, which is expressed on macrophages, NK cells, and dendritic cells, recognizes classical and nonclassical MHC class I molecules on tumor cells to suppress phagocytosis through a mechanism that is analogous to that of CD47–SIRPα, whereas other family members, including LILRB2 and LILRB4, regulate macrophage polarization, dendritic cell tolerance, and MDSC-mediated immune suppression (65, 66). More generally, the Siglec family of sialic acid-binding immunoglobulin-like lectins, which includes both Siglec-10 and Siglec-15, contributes additional layers of myeloid- and T-cell-directed suppression. Specifically, Siglec-15 is primarily upregulated on tumor cells and tumor-infiltrating myeloid cells in a manner that is mutually exclusive with PD-L1 expression, thereby positioning it as a candidate checkpoint in tumors that are less responsive to PD-1/PD-L1-directed therapy (67).

Collectively, these pathways, together with CD200–CD200R, indicate that innate immune checkpoints are not redundant but instead operate through distinct signaling axes that can be co-expressed within the same tumor microenvironment. This scenario raises the possibility that the combined targeting of CD200–CD200R with CD24–Siglec-10, LILRB-family, or Siglec-15 blockade could achieve more complete relief of myeloid-mediated immunosuppression than the inhibition of any single checkpoint alone, particularly in tumors in which multiple “don’t eat me” or myeloid-inhibitory signals are co-expressed. However, because these pathways engage overlapping but nonidentical downstream signaling machinery, as well as the fact that CD200–CD200R itself exerts effects that extend beyond phagocytosis to encompass broader myeloid activation and cytokine production, rational combination strategies will require systematic preclinical characterizations of ligand and receptor co-expression patterns, assessments of additive versus synergistic efficacy, and careful monitoring of cumulative toxicity, particularly given the physiological roles that several of these pathways play in maintaining immune tolerance outside of the tumor setting.

## Therapeutic opportunities and tumor-type-specific strategies

### CD200–CD200R blockade as a myeloid immune checkpoint therapy

**Figure 2:**
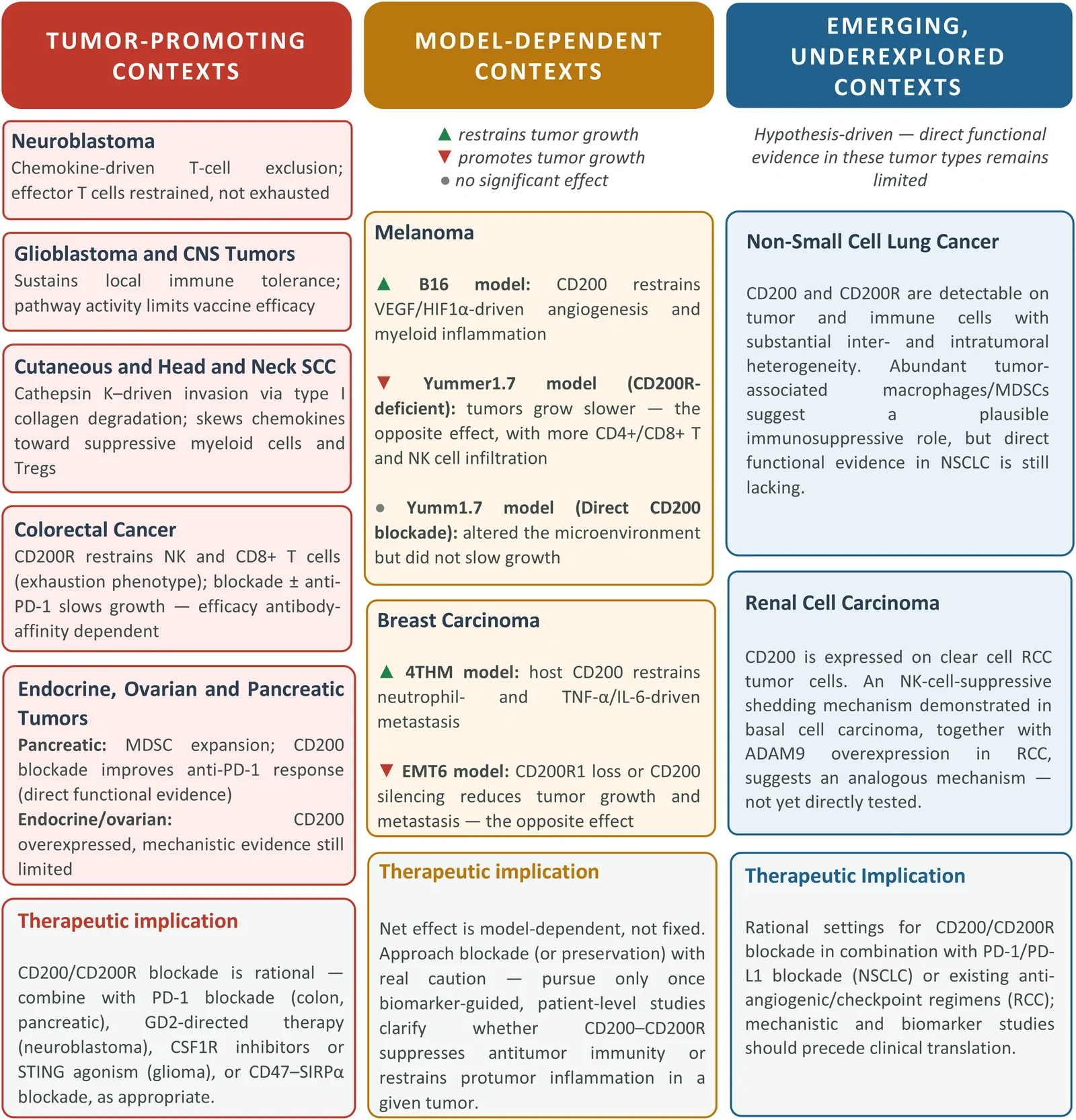
Context-dependent roles of the CD200–CD200R axis across solid tumors. The biological and therapeutic effects of the CD200–CD200R axis vary substantially across solid tumor types and can be divided into three categories. A predominantly protumor role (left) is supported in neuroblastoma (chemokine-mediated exclusion of effector T cells), glioblastoma and other CNS tumors (local immune tolerance and reduced vaccine efficacy), as well as cutaneous and head and neck squamous cell carcinomas (cathepsin K-mediated collagen degradation and tumor invasion), colorectal cancer (CD200R-mediated NK-cell and CD8^+^ T-cell dysfunction, reversible with CD200R blockade alone or combination with PD-1 blockade), and several endocrine, ovarian, and pancreatic malignancies, among which direct functional evidence of reduced MDSCs and enhanced anti-PD-1 responses has been reported specifically in pancreatic cancer. These findings support the evaluation of CD200–CD200R-targeted therapies combined with PD-1 blockade, anti-GD2 therapy, CSF1R inhibitors, STING agonists, or CD47–SIRPα-targeted therapies. Melanoma and breast carcinoma (center) instead demonstrate model-dependent (rather than uniform) biological effects; specifically, in B16 melanoma and 4THM breast carcinoma models, CD200–CD200R signaling suppresses protumor myeloid inflammation, angiogenesis, and neutrophil- and cytokine-mediated metastatic progression, whereas in CD200R-deficient-host Yummer1.7 melanoma and CD200R1-deficient or CD200-silenced EMT6 breast carcinoma models, the loss of CD200–CD200R signaling instead reduces tumor growth and metastasis; additionally, direct CD200 blockade in the Yummer1.7 melanoma model altered the immune microenvironment without measurable antitumor activity. These findings argue against indiscriminate inhibition or preservation of the pathway in melanoma and breast carcinoma; instead, they support biomarker-guided, patient-specific decision-making. Non-small cell lung cancer (NSCLC) and renal cell carcinoma (RCC) (right) represent a third emerging category; specifically, CD200/CD200R are expressed with substantial heterogeneity across NSCLC tumors, and CD200 is expressed on clear cell RCC tumor cells with a plausible but untested NK-cell-suppressive shedding mechanism; moreover, direct functional evidence in both tumor types remains limited, thereby positioning these tumors as high-priority contexts for future investigation, potentially combined with PD-1/PD-L1 blockade (NSCLC) or existing antiangiogenic/checkpoint regimens (RCC).

Preclinical studies in neuroblastoma and squamous cell carcinoma models have shown that inhibition of CD200 or CD200R reduces immunosuppressive myeloid infiltrates, enhances effector T-cell infiltration, and suppresses tumor progression (12, 45). In glioblastoma models, CD200 blockade similarly augments vaccine-induced T-cell responses, supporting its potential as an immunotherapeutic adjunct (31). Clinical translation of this strategy is already underway. The anti-CD200 antibody samalizumab has been evaluated in patients with hematologic malignancies (10), while the CD200R1-blocking antibody 23ME-00610 has completed a first-in-human phase 1 study in patients with advanced solid tumors. The phase 1 trial demonstrated an acceptable safety profile, dose-dependent target engagement, and high tumor CD200 expression as a potential predictive biomarker of therapeutic benefit (17). Collectively, these findings support continued clinical development of CD200- and CD200R-targeted therapies for solid tumors. However, the context-dependent biology discussed throughout this review indicates that therapeutic strategies should be tailored to individual tumor types rather than applied uniformly across solid malignancies (**Figure 2**).

### Combination strategies with T-cell immune checkpoint inhibitors

Because the CD200–CD200R axis primarily regulates the myeloid compartment while indirectly shaping T-cells priming and activation, combining CD200 pathway modulation with PD-1/PD-L1 or CTLA-4 blockade represents a logical therapeutic strategy, particularly in tumors that respond poorly to T-cell checkpoint inhibition alone (1, 2). Such combination approaches may be especially valuable in neuroblastoma, squamous cell carcinomas, and ovarian cancer, where alleviating myeloid-mediated immunosuppression could enhance sensitivity to T-cell-directed immunotherapies. Pancreatic cancer provides direct preclinical support for this strategy. In experimental models, CD200 blockade reduces intratumoral MDSCs and significantly enhances the antitumor efficacy of PD-1 blockade (52).

### Combination strategies with CD47–SIRPα inhibitors and myeloid-reprogramming agents

Given its emerging role as a phagocytosis checkpoint, the CD200R1–CD200 axis is a logical partner for therapies targeting CD47–SIRPα. Concurrent inhibition of these pathways has been shown to enhance macrophage-mediated tumor cell clearance beyond the effects achieved by either strategy alone (9). Combining these approaches with myeloid-reprogramming agents, such as CSF1R inhibitors, which have demonstrated activity in glioma and other solid tumors (43, 68), or stimulator of interferon genes (STING) agonists, which induce robust systemic antitumor immunity (69, 70), may further promote the activation of macrophages and dendritic cells into antitumor effector states (71).

Several tumor-specific combination strategies warrant further investigation. In glioma, CD200 blockade could be combined with therapeutic vaccines and STING agonists, with microglial activation and T-cell infiltration serving as key pharmacodynamic endpoints. In squamous cell carcinoma, chemoradiotherapy could be combined with dual CD200R1 and CD47 blockade together with CSF1R inhibition, while monitoring changes in invasive behavior and myeloid cell phenotype. For neuroblastoma, combining anti-GD2-based therapy with CD200R1 inhibition and PD-1 blockade represents another promising strategy (42).

### Cautious, model-informed therapeutic use in melanoma and breast carcinoma

As discussed above, the biological effects of the CD200–CD200R axis in melanoma (12–14, 37) and breast carcinoma (39, 40) are highly model dependent rather than uniformly protumorigenic or tumor suppressive, with divergent effects observed across experimental models. Consequently, indiscriminate blockade or enhancement of this pathway may either exacerbate pathogenic myeloid-driven inflammation or eliminate a biologically relevant tumor-suppressive immune response, depending on the underlying tumor context. Accordingly, therapeutic modulation of the CD200 pathway in melanoma and breast carcinoma should be approached with caution. Clinical application should await biomarker-guided studies capable of determining, on an individual patient basis, whether CD200–CD200R signaling predominantly suppresses protective antitumor immunity or restrains tumor-promoting inflammatory processes within a given tumor. A patient-specific strategy will be essential across both malignancies to define the predominant role of this signaling axis before it can be therapeutically targeted.

## Biomarkers and patient selection in solid tumors

Successful clinical implementation of CD200–CD200R-targeted therapies will depend on identifying the patients most likely to benefit. Multiplex immunohistochemistry and spatial expression profiling have begun to characterize the distribution of CD200 and CD200R across tumor and stromal compartments in pancreatic (54) and lung cancer cohorts (57). However, both studies reported substantial heterogeneity in tissue CD200 expression, and neither found tissue expression to be an independent prognostic factor. Emerging evidence suggests that circulating biomarkers may provide additional clinical value. In pancreatic cancer, elevated plasma concentrations of sCD200, rather than tissue CD200 expression, have been associated with significantly poorer overall survival and progression-free survival (72). These findings indicate that circulating sCD200 may better reflect systemic CD200–CD200R pathway activity than tissue expression alone and therefore warrants further evaluation as a predictive or pharmacodynamic biomarker.

Several biological features may help explain why circulating sCD200, rather than CD200 expression in tumor tissue, appears to be a more robust biomarker in pancreatic ductal adenocarcinoma. First, pancreatic tumors are characterized by a dense desmoplastic stroma and spatially heterogeneous CD200 expression within the tumor epithelium; thus, a single tissue biopsy may not adequately capture the overall CD200 burden across the lesion, whereas circulating sCD200 integrates shedding from the entire tumor mass and its associated stroma. Second, plasma sCD200 levels reflect proteolytic shedding by matrix metalloproteinases and other sheddases that are frequently upregulated within the pancreatic tumor microenvironment; thus, elevated sCD200 levels may function as a surrogate marker of overall CD200–CD200R pathway activity, including contributions from tumor, stromal, and MDSC-associated sources, that static tissue immunohistochemistry cannot fully capture. Third, because shed sCD200 retains the capacity to systematically engage CD200R, including on circulating monocytes and MDSCs, it may more directly be able to track functional pathway engagement and immunosuppressive potential than membrane-bound tumor CD200 expression, which does not necessarily indicate active receptor engagement. Finally, pancreatic ductal adenocarcinoma is frequently associated with substantial circulating tumor burden and systemic inflammation, and these conditions are associated with the accumulation of shed sCD200, thus providing a more dynamic, minimally invasive, and serially measurable readout of disease activity than a fixed tissue expression pattern assessed at a single time point. Together, these features may explain why elevated plasma sCD200 levels, rather than tissue CD200 expression, are specifically associated with poorer overall survival and progression-free survival in patients with pancreatic ductal adenocarcinoma (72).

The relative utility of tissue versus circulating CD200 as a biomarker is also expected to vary by tumor type, thereby reflecting differences in tumor accessibility, shedding biology, and the extent to which CD200 expression is tumor-intrinsic versus systemically driven. **Table 1** summarizes the biomarker modality that is most supported by the current evidence for each tumor type discussed in this review.

**Table 1:** Tissue CD200 versus circulating soluble CD200 (sCD200) as the more appropriate biomarker by tumor type, based on the evidence discussed in this review.

Tumor type	Preferred biomarker modality	Rationale
Pancreatic cancer	Circulating sCD200 (plasma)	Elevated plasma sCD200, rather than tissue CD200 expression, is associated with poorer overall and progression-free survival; better reflects systemic pathway activity and MDSC engagement (54, 72).
Non-small cell lung cancer	Tissue CD200/CD200R (spatial profiling)	Substantial intratumoral heterogeneity in tissue expression has been reported; a circulating biomarker has not yet been validated in this tumor type (57).
Glioblastoma and other CNS tumors	Circulating sCD200 (serum or cerebrospinal fluid)	Circulating sCD200 correlated with MDSC expansion, disease progression and recurrence, making it a more functionally relevant biomarker of CD200-mediated immunosuppression (31).
Melanoma	Tissue CD200 (tumor-intrinsic expression)	Biological effect is model- and context-dependent and tied mechanistically to tumor-derived CD200 expression rather than to systemic shedding (12–14, 37).
Breast carcinoma	Tissue CD200 (tumor- and host-derived)	Divergent, context-dependent effects arise from the interplay of tumor- and host-derived CD200 and CD200R1, best captured at the tissue level (39, 40).
Neuroblastoma	Tissue CD200 (tumor overexpression)	Tumor-intrinsic CD200 overexpression drives local chemokine remodeling and T-cell exclusion within the tumor microenvironment (12, 41).
Cutaneous and head and neck squamous cell carcinoma	Tissue CD200 with myeloid infiltration	Local invasion is driven by CD200-dependent myeloid reprogramming; tissue-based markers of myeloid infiltration may best reflect this mechanism (45–48).
Colorectal cancer	Tissue CD200 (tumor expression)	Therapeutic efficacy of CD200R blockade has been linked to tumor CD200 expression and antibody potency, supporting tissue-based patient selection (50, 51).
Endocrine and ovarian malignancies	Tissue CD200 (tumor expression)	Mechanistic and biomarker data remain limited relative to other tumor types; CD200 is expressed in a high proportion of neuroendocrine neoplasm and ovarian carcinoma tissue samples (53, 55).
Renal cell carcinoma	Tissue CD200 (with ADAM9 co-expression as current foundation)	CD200 expression has been demonstrated on clear cell RCC tumor cells; but currently underexplored in the context of CD200-directed biomarker (55, 60).

CNS, central nervous system; MDSC, myeloid-derived suppressor cell.

Expression profiling alone is unlikely to be sufficient for patient stratification and should be complemented by functional and systemic biomarkers of CD200–CD200R pathway activity. Functional assessment of the myeloid compartment—including ex vivo assays of macrophage phagocytosis, dendritic cell antigen presentation, and myeloid cytokine production—may provide direct insight into the extent of CD200-mediated immune suppression within individual tumors. These approaches build on established methodologies for evaluating MDSC function and macrophage polarization (33, 73, 74). Circulating sCD200 may provide an additional minimally invasive biomarker of pathway activity. Measurement of sCD200 in serum or cerebrospinal fluid could be particularly informative in CNS tumors and other malignancies characterized by high levels of CD200 shedding (31).

## Outstanding questions

Several important questions remain regarding the therapeutic application of the CD200–CD200R axis in solid tumors. A major unresolved issue is identifying the tumor contexts in which CD200–CD200R signaling primarily suppresses protective antitumor immunity versus those in which it restrains tumor-promoting inflammation and determining how these opposing functions can be reliably distinguished in human tissues rather than inferred from preclinical mouse models (12–14, 37). This challenge is particularly evident in melanoma and breast carcinoma, where conflicting findings have been reported across experimental models of the same tumor type (12–14, 37, 39, 40). Consequently, it remains uncertain which preclinical models most accurately reflect the biological role of CD200–CD200R signaling in human disease.

Additional questions concern the relative contributions of different cellular sources of CD200. The interactions among tumor cells, stromal cells, and normal parenchymal tissues remain poorly defined in melanoma (37), neuroblastoma (41), CNS tumors (31), and squamous cell carcinomas (45). Likewise, the respective roles of membrane-bound and sCD200 in regulating immune suppression across these tumor types require further investigation (20). In colorectal cancer, another key question is the therapeutic threshold required for effective CD200R blockade. Future studies should clarify how antibody affinity, dosing, and tumor CD200 expression influence treatment efficacy, particularly in light of findings showing that genetic *Cd200r* deletion and high-affinity CD200R-blocking antibodies produced robust antitumor effects, whereas a lower-affinity antibody did not (50, 51).

The optimal strategy for targeting the CD200–CD200R axis remains to be established, including whether inhibition of CD200 or CD200R offers greater therapeutic benefit. Equally important is determining whether tumor-specific approaches can enhance antitumor immunity while preserving the physiological immune tolerance required for tissue homeostasis, particularly in the CNS and skin (32).

Another unresolved issue is how best to integrate CD200–CD200R-targeted therapies with other CD47–SIRPα, PD-1/PD-L1, CTLA-4, and myeloid-reprogramming agents within rational combination regimens for solid tumors (3, 17).

Finally, the most informative clinical trial designs remain to be defined. Promising strategies include vaccine-based combination trials in glioblastoma, anti-GD2-based immunotherapy combined with immune checkpoint blockade in neuroblastoma, and chemoradiotherapy-based combination regimens in squamous cell carcinoma. In NSCLC and RCC, for which direct mechanistic evidence remains limited, priority should instead be given to mechanistic and spatial-profiling studies confirming CD200–CD200R pathway activity, followed by early-phase combination trials with PD-1/PD-L1 blockade in NSCLC and with existing antiangiogenic/checkpoint regimens in RCC. Determining which of these approaches most effectively demonstrate the clinical benefit of CD200–CD200R modulation will be essential for guiding future therapeutic development.

## Conclusions

The CD200–CD200R axis is a myeloid-centered immune checkpoint whose biological and therapeutic effects are highly context dependent and, in some tumor types, vary even across preclinical models. In neuroblastoma, glioblastoma, squamous cell carcinomas, colorectal cancer, and several endocrine, ovarian, and pancreatic malignancies, CD200–CD200R signaling suppresses the activation of macrophages, dendritic cells, NK cells, and CD8+ T cells, impair phagocytosis and antigen presentation, and promotes immunosuppressive cellular infiltrates that facilitate tumor immune evasion (31, 41, 45, 50–55). In contrast, evidence from melanoma and breast carcinoma indicates that the biological effects of CD200–CD200R are model dependent rather than uniformly protumorigenic or tumor suppressive. In some experimental models, tumor cell-derived CD200 limits protumor myeloid inflammation and metastatic progression, whereas in others, CD200R-deficient hosts or CD200-silenced tumors exhibit reduced tumor growth and metastasis. Moreover, direct evaluation of CD200 blockade in melanoma has not consistently demonstrated therapeutic benefit (13, 14, 37, 39, 40). Collectively, these findings emphasize that the role of CD200–CD200R in these tumor types cannot be generalized and should instead be interpreted within the context of the specific experimental model and tumor biology (12) (**Figure 2**).

These divergent biological functions indicate that the CD200–CD200R axis should not be regarded as a universally actionable therapeutic target. Instead, its clinical application should be guided by a context-dependent framework that considers tumor type, tumor microenvironment architecture, and the dominant myeloid and lymphoid immune programs. Integrating mechanistic insights with spatial and functional biomarker profiling and tumor-specific clinical trial design will facilitate the rational development of CD200–CD200R-targeted therapies. Within this precision immunotherapy framework, the CD200–CD200R axis can be incorporated alongside CD47–SIRPα and other emerging myeloid immune checkpoints to reprogram immunosuppressive tumor microenvironments in settings where the underlying biology supports therapeutic intervention (9, 10, 12, 17, 54, 75).
